# Supplementary material for: MetaRibo-Seq measures translation in microbiomes
Source: Nat Commun. 2020 Jun 29;11:3268. doi: 10.1038/s41467-020-17081-z (PMC7324362; doi:10.1038/s41467-020-17081-z)
Supplement: Supplementary file 10 — Supplementary Data 7 [file 41467_2020_17081_MOESM10_ESM.zip › File2/Confidence_High_Taxonomy/379785_out.krona.html]

Javascript must be enabled to view this page.

members
magnitude
magnitudeUnassigned
count
unassigned
taxon
rank

379785\_out

2

2
2
superkingdom

976
1
phylum

class
200643
1

order
1
171549

815
1
family

genus

SRS017859\_contig\_number\_4202
816
1

1
1239
phylum

1
186801
class

order
186802
1

family
1
186803

1
841
genus

species

SRS016335\_contig\_number\_contig-100\_1227.238748
360807
1
